# Rapid time-resolved Circular Polarization Luminescence (CPL) emission spectroscopy

**DOI:** 10.1038/s41467-020-15469-5

**Published:** 2020-04-03

**Authors:** Lewis E. MacKenzie, Lars-Olof Pålsson, David Parker, Andrew Beeby, Robert Pal

**Affiliations:** Department of Chemistry, Durham University, South Road, Durham, DH1 3LE UK

**Keywords:** Analytical chemistry, Photochemistry, Excited states, Optical spectroscopy, Optical spectroscopy

## Abstract

Circular polarisation luminescence (CPL) emission spectroscopy is a powerful tool for probing the fundamental chiroptical features of optically emissive chiral molecular systems. However, uptake of CPL spectroscopy has been impeded by the limitations of conventional scanning monochromator (SM) CPL spectrometers, which are costly to acquire and maintain, and typically require tens of minutes to acquire a typical CPL spectrum. Here, we demonstrate a design of CPL spectrometer which uses rapid readout solid state (SS) spectrometer detectors and a dual channel optical layout to acquire CPL spectra in as little as 10 milliseconds. We validate and demonstrate equivalent CPL measurement by measuring CPL spectra of two reference europium(III) complexes. Further, we demonstrate time-gated CPL acquisition, enabling long-lived CPL luminescence to be distinguished from short-lived emission of other fluorescent species. We anticipate that SS-CPL spectrometers will enable flexible, rapid, and relatively low-cost CPL spectroscopy for diverse applications.

## Abstract (web summary)

Circular polarization luminescence (CPL) spectroscopy is a tool to study chiroptical systems, but the measurement process is generally very slow. The authors introduce a CPL technique with much faster acquisition, demonstrating meaningful time-dependent measurements and enabling new applications.

## Introduction

Chirality in optically emissive molecular systems can manifest as the emission of left or right circularly polarised luminescence (L-CPL, R-CPL), corresponding to photons of spin angular momentum $+ \hbar$ and $- \hbar$, respectively^1–3^. Circularly polarised luminescence (CPL) spectroscopy provides fundamental insight into the stereochemical structures and associated transitions of chiral molecules; CPL emission is maximised for electronic transitions that are predominately magnetic dipole allowed^4,5^. CPL emission is quantified in absolute terms by the emission dissymmetry factor: $g_{{\mathrm{em}}} = {\textstyle{{2\left( {I_{{\mathrm{L}} {\hbox{-}} {\mathrm{CPL}}} - I_{{\mathrm{R}} {\hbox{-}} {\mathrm{CPL}}}} \right)} \over {\left( {I_{{\mathrm{L}} {\hbox{-}} {\mathrm{CPL}}} + {\mathrm{I}}_{{\mathrm{R}} {\hbox{-}} {\mathrm{CPL}}}} \right)}}}$, where *I*_L-CPL_ and *I*_R-CPL_ are the intensities of L-CPL and R-CPL light emitted. Thus, a value of *g*_em_ = 2 indicates 100% L-CPL emission, and *g*_em_ = −2 indicates 100% R-CPL emission. A wide variety of CPL emitting molecular systems have been reported (see Table 1)^5–18^, with lanthanide complexes best suited to producing strong CPL emission ($\left| {g_{{\mathrm{em}}}} \right|$ up to ~1.38 reported)^19^. CPL emitting molecular systems have been proposed for diverse applications, including cellular imaging^20,21^, molecular biosensing^21–24^, display technologies^25–29^, and as advanced security inks^30^.

**Table 1:** Representative maximum $\left| {g_{{\mathrm{em}}}} \right|$ for various chiral molecular systems.

Chiral system	Representative maximum *g*_em_
Lanthanides^5,10,22–25,27,33,36–38,43,44,49,61^	~10^−1^
Helicenes^5,6^	~10^−2^
Ketones^5^	~10^−2^
Helical polymers^11–14^	~10^−2^
BODIPYs^15,16^	~10^−3^
Self-assembled chiral nanoparticles^17^	~10^−3^
Quantum dots^7,18^	~10^−3^
Nanographene^8^	~10^−4^
Proteins^9^	~10^−4^

For over 50 years^31,32^, CPL spectrometers have typically been custom-built, utilising a photoelastic modulator (PEM) and scanning monochromators (SMs) to provide chiroptical and spectral discrimination^33^. A PEM functions as an electronically switchable quarter wave plate (QWP) oscillating at radio frequencies to convert L-CPL and R-CPL light to orthogonal linearly polarised states, which are subsequently analysed by a static linear polariser. The intensity of the resultant radio-frequency oscillating signal is quantified by a photodetector with the aid of a lock-in amplifier. However, SM-CPL spectrometers have two main drawbacks: (1) the considerable expense of constructing and maintaining instrumentation; and (2) slow scan rates, requiring several tens of minutes to acquire a single CPL scan spectra, e.g. ~45 min (for a typical 150 nm scan range with 0.5 nm sampling steps and 500 μs integration time over 5 accumulations).

In recent years, the advent of low-cost, rapid read-out, miniature solid state (SS) spectrometers using solid state detector arrays (e.g., charge coupled devices (CCDs) or complimentary metal-oxide semiconductors (CMOS)) have enabled convenient, rapid, whole-spectrum measurements in as little as a few milliseconds, enabling widespread and diverse spectroscopy applications^34,35^.

**Fig. 1:**
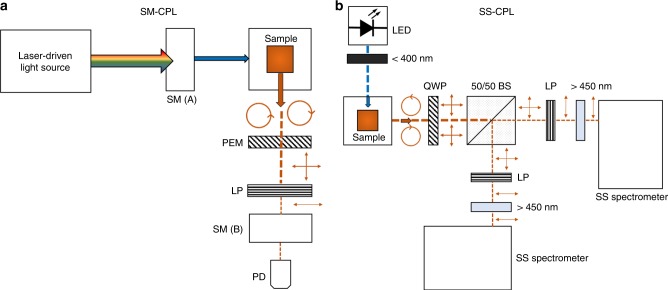
Optical layouts of the CPL spectrometers. Circles with arrows represents circularly polarised light; vertical and horizontal arrows represent linearly polarised light. **a** SM-CPL spectrometer. Laser driven light source. SM(A) = excitation scanning monochromator; PEM = photoelastic modulator (effectively acting an electrically switched variable quarter wave plate operating at radio frequencies); LP = linear polariser; SM(B) emission scanning monochromator; PD = photodiode. **b** SS-CPL spectrometer. LED = light emitting diode (emission peak at 365 nm); <400 nm = short pass filter; QWP = quarter wave plate; 50/50 BS = 50/50 non-polarising beam splitter; LP = linear polariser; >450 nm = long pass filter; SS spectrometer = solid state CCD spectrometer.

Herein, we report a rapid CPL spectroscopy by combining SS spectrometers with an elegant and novel dual-channel optical layout (see Fig. 1 and Supplementary Fig. 1) to acquire CPL spectra in as little as 10 milliseconds. In the SS-CPL spectrometer, a static achromatic quarter wave plate (QWP) converts L-CPL and R-CPL light to orthogonal linearly polarised light, which is subsequently split into two spatially separated detection channels by a beam splitter. Each detection channel can independently analyse both L-CPL and R-CPL states by automated rotation of a linear polariser prior to whole-spectra detection with a SS 2DCCD spectrometer. Simultaneous operation of both channels enables whole-spectra CPL acquisition in real time. We demonstrate that the SS-CPL spectrometer measures CPL spectra equivalent to a conventional SM-CPL spectrometer (previously used in many published studies) by measuring CPL emission from two europium(III) reference complexes. Further, we demonstrate time-gated CPL detection with the SS-CPL spectrometer. We anticipate that the advantages of versatility, repeated rapid scan acquisition, and affordability offered by SS-CPL spectrometers will promote CPL spectroscopy, which has been relatively unexploited in comparison to its absorption analogue, circular dichroism.

## Results

### Comparison of SM-CPL and SS-CPL spectrometer performance

CPL spectra of two CPL reference samples were measured using both a new SS-CPL spectrometer and a SM-CPL spectrometer that had been used in many prior studies (see Fig. 1 and the Methods section for full technical workings of both CPL spectrometers)^21,22,24,36–41^. The first reference sample was 5.5 mM tris(3-trifluoro-acetyl-(+)-camphorato)europium(III) ([Eu{(+)-facam}_3_]) in anhydrous DMSO (see Supplementary Fig. 4 for structure). The use of Eu{(+)-facam}_3_ as a standard reference sample for CPL spectrometers has been reported by others: it is commercially available and displays strong feature-rich CPL emission, particularly in the ^5^D_0_→^7^F_1_ transition band (*g*_em_ ~ −0.78)^5,32^. However, only a single enantiomer of Eu{(+)-facam}_3_ is commercially available. The second CPL reference sample was Eu·L^1^, a complex which has recently been developed in house to serve as a dependable low-energy reference standard for CPL spectrometers^42^. Eu·L^1^ is a derivative of *N*,*N*′-bis(1-phenyl-ethyl)-2,6-pyridinecarboxamide ([Eu:BPEPC]), a CPL reference standard complex previously proposed by others for several reasons: (1) access to two enantiomers; (2) strong and intricate CPL emission across both enantiomers ($\left| {g_{{\mathrm{em}}}} \right|\;{\mathrm{\sim 0}}{\mathrm{.2}}$ for the ^5^D_0_→^7^F_1_, ^5^D_0_→^7^F_2,_ and ^5^D_0_→^7^F_3_ transitions); (3) demonstrated stability in solution over extended time-periods, and resistance to photobleaching^5,43,44^. However, EU:BPEPC requires high energy excitation, necessitating use of specialist 308 nm laser excitation and a relatively high concentration of ~6.6 mM^5,43,44^. This renders EU:BPEPC incompatible with our SS-CPL spectrometer, which incorporates a 365 nm light emitting diode (LED) as an excitation light source. Eu·L^1^ (see Supplementary Fig. 4 for structure) was designed to preserve the favourable CPL emission properties of Eu:BPEPC, whilst enabling efficient excitation at lower energy wavelengths (i.e., 365 nm). Full details of the synthesis and photophysical properties of Eu·L^1^ were recently published by Starck et al.^42^.

**Fig. 2:**
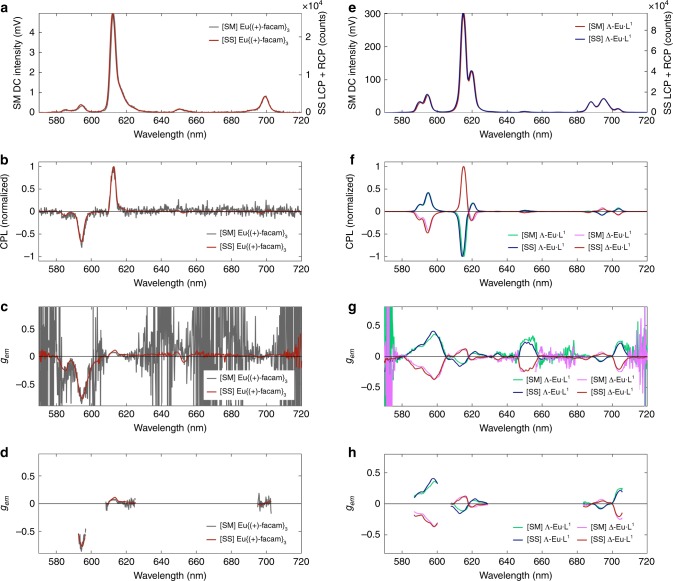
Close agreement between SM-CPL and SS-CPL spectrometers demonstrated by measuring CPL of two reference complexes. 5.5 mM Eu{(+)-facam}_3_ in anhydrous DMSO: **a** Total intensity, **b** CPL, **c** raw *g*_em_, **d** *g*_em_ (threshold cut-off 0.22 mV [SM-CPL total intensity]). 18 µM Eu·L^1^ dissolved in a 50:50 mixture of MeCN and MeOH: **e** Total Intensity. **f** CPL, **g** raw *g*_em_, **h** thresholded *g*_em_ (threshold cut-off 4 mV [SM-CPL total intensity]). Both samples were excited at 365 nm. See the Methods section for scan settings.

The CPL spectra recorded by the SM-CPL and SS-CPL spectrometers for Eu{(+)-facam}_3_ and Eu·L^1^ show close agreement in terms of total intensity, CPL, and recovered *g*_em_ across all emission wavebands (see Fig. 2). For 18 µM Eu·L^1^ dissolved in 50:50 MeCN and MeOH, all CPL transition features are closely replicated in both the SM and SS CPL spectrometers, with similarly close matches achieved in estimates of *g*_em_ at the peak of transitions (Fig. 2e–h). Despite the overall bright emission of Eu·L^1^, estimates of *g*_em_ for relatively low emission intensity wavebands (e.g., ~650 nm) were prone to noise when measured with the SM-CPL spectrometer. In contrast, the SS-CPL spectrometer CPL spectra was less prone to measurement noise due to superior excitation efficiency and the ability to average many spectra rapidly acquired in succession (see Fig. 2g). For display clarity, wavebands of very low or no emission (SM-CPL total intensity < 4 mV, i.e., 1.3% of maximum total emission intensity) were thresholded from *g*_em_ estimates (see Fig. 2h). Some minor discrepancies in estimated *g*_em_ can be observed at wavelengths where the rate of change of total intensity measurements versus wavelength is greatest (Fig. 2h); these discrepancies were due to the differing instrumental line profiles of the two CPL spectrometers, which could not be precisely matched owing to the fundamentally different spectral discrimination modalities used by the two CPL spectrometers (i.e., scanning monochromators vs. solid state CCD spectrometers detectors). However, these minor *g*_em_ spectra discrepancies are not problematic because *g*_em_ is not conventionally reported as a continuous spectra, rather it is reported as a single number relating to the peak wavelength of each transition waveband, where excellent agreement (within experimental errors) between the SS-CPL and SM-CPL spectrometers was achieved. Overall, the measurements of Eu·L^1^ demonstrate close agreement between the SS-CPL and SM-CPL spectrometers.

Measurement of the CPL spectra of 5.5 mM Eu{(+)-facam}_3_ in anhydrous DMSO enables comparison to *g*_em_ values for a near identical sample reported independently by Sánchez-Carnerero et al.)^5^. First, it is necessary to note the relatively low emission intensity recorded by the SM-CPL spectrometer for Eu{(+)-facam}_3_ in comparison to the strong the emission intensity recorded for 18 µM Eu·L^1^ (~5 mV vs. ~300 mV recorded by the SM-CPL respectively). This low total emission intensity is a problematic characteristic of Eu{(+)-facam}_3_; resulting in sub-optimal signal to noise ratio (SNR) for CPL measurements (see Fig. 2b, c). The SS-CPL spectrometer recorded a higher SNR than the SM-CPL spectrometer because the SS-CPL spectrometer is capable of rapid spectral acquisition, enabling the accumulation and averaging of many spectra to increase SNR (${\mathrm{SNR}} \propto \root {2} \of {N}$, where *N* is the number of scans). The SS-CPL spectrometer is aided by a dedicated 365 nm LED illumination (modular and interchangeable to any desired light source and wavelength in future), which provides ample excitation for Eu{(+)-facam}_3_. SNR was estimated fitting a Gaussian profile to the CPL peaks associated with a given transition. For the ^5^D_0_→^7^F_1_ transition (595.2 nm) SNR recovered by the SM-CPL was 18, whereas SNR recovered by the SS-CPL spectrometer was 148. For the SM-CPL spectrometer to yield a similar SNR to the SS-CPL spectrometer via signal averaging would require 64 more repeated scans; assuming standard scan settings, this would require at least 48 h of constant scanning with the SM-CPL spectrometer. This would pose serious limitations and would likely contribute to irreversible photodegradation of the sample. For SS-CPL spectrometer measurements, the uncertainty of emission dissymmetry factor, Δ*g*_em_, was directly calculated from the standard error of intensity recorded by many consecutive measurements of *I*_L-CPL_ and *I*_R-CPL_ (see Supplementary Note 3). For many studies utilising SM-CPL spectrometer measurements, a nominal instrumental uncertainty of Δ*g*_em_ ± 0.01 is typically reported. However, this nominal instrumental SM-CPL spectrometer uncertainty assumes a high SNR for total intensity and CPL measurements by the SM-CPL spectrometer, an assumption that does not hold for the measurement of Eu{(+)-facam}_3_ achieved here. Instead, we calculated uncertainty in Δ*g*_em_ of Eu{(+)-facam}_3_ by first estimating SNR for a transition by fitting a curve to both CPL and total intensity transitions (SNR = 1/fractional uncertainty). The *g*_em_ estimate for the ^5^D_0_→^7^F_1_ waveband acquired by the SS-CPL spectrometer agrees with the value reported Sánchez-Carnerero et al.^5^ to within 2%, i.e. *g*_em(595.2 nm)_ = −0.763 ± 0.018 (2.4%) and *g*_em(595.2 nm)_ = −0.78± 0.01 (1.3%) respectively (see Table 2); this is an excellent agreement considering experimental variabilities such as sample preparation and differences in CPL spectrometer detection modalities and scan settings. However, for the same transition, the SM-CPL spectrometer estimated *g*_em(595.2 nm)_ = −0.70 ± 0.06 (9%); the high uncertainty is due to poor SNR, yet the measurement agrees with the SS-CPL spectrometer to within uncertainty limits and is also close to the value, *g*_em(595.2 nm)_ = −0.7 8± 0.01 (1.3%), reported by Sánchez-Carnerero et al.^5^. It should be noted that in 1976 Brittain and Richardson^32^ reported a representative *g*_em_ for the ^5^D_0_→^7^F_1_ (peak at 595.5 nm) emission band of −0.84 with no uncertainty quoted^32^. This is significantly larger than the more recent reference value reported by Sánchez-Carnerero et al.^5^. However, it is not clear whether or not Brittain and Richardson simply reported maximum *g*_em_ for the ^5^D_0_→^7^F_1_ emission waveband, or more specifically *g*_em_ at 595.5 nm^32^. This highlights a current ambiguity of inter-study and inter-instrument ‘ground truth’ measurements with Eu{(+)-facam}_3_. For the ^5^D_0_→^7^F_2_ waveband, Sánchez-Carnerero et al. reported *g*_em(613.5 nm)_ = +0.07 ± 0.01 (14%), which matches the value recovered by the SM-CPL spectrometer exactly; *g*_em_ recovered from the SS-CPL spectrometer agrees less closely *g*_em(613.5 nm)_ = +0.114 ± 0.018 (16%). However, it should be noted that both the SM-CPL and SS-CPL spectrometers recover maximum CPL emission at 612.7 nm, where the SM-CPL spectrometer recovers *g*_em_ = +0.07 ± 0.01 and the SS-CPL recovers *g*_em(612.7 nm)_ = 0.100 ± 0.018, i.e., agreeing within experimental uncertainties. These minor inter-study discrepancies serve to highlight the aforementioned importance of instrumental line profile in CPL spectroscopy because it influences emission line width and line shape, consequently affecting the position and the intensity of spectral emission features. Overall, the measurements of Eu{(+)-facam}_3_ demonstrate inter-study and inter-instrument agreement, and further demonstrate the advantages offered by the SS-CPL spectrometer, i.e., efficient excitation and rapid readout/detection of CPL spectra.

**Table 2:** Experimental *g*_em_ values for 5.5 mM Eu{(+)-facam}_3_ in anhydrous DMSO versus those obtained by reported by Sánchez-Carnerero et al.^5^.

Transition (wavelength)	*g*_em_ (Sánchez-Carnerero et al.)	*g*_em_ (SM-CPL)	*g*_em_ (SS-CPL)
^5^D_0_→^7^F_1_ (595.2 nm)	−0.78 ± 0.01 (1.3%)^a^	−0.70 ± 0.06 (9%)^b^	−0.763 ± 0.018 (2.4%)^c^
^5^D_0_→^7^F_2_ (613.5 nm)	+0.07 ± 0.01 (14%)^a^	+0.07 ± 0.01 (14%)^a^	+0.114 ± 0.018 (16%)^c^

^a^Nominal SM-CPL instrument uncertainty (assumed).

^b^Uncertainty calculated from SNR estimated by fitting Gaussian curves to CPL and total intensity measurements using least mean squares regression.

^c^Uncertainty directly calculated from standard error of the mean *I*_L-CPL_ and *I*_R-CPL_ from 20 repeated measurements.

### Time-gated whole-spectrum SS-CPL spectroscopy

Time-gated CPL measurement is desirable to separate long-lived emission (e.g., that of enantiopure lanthanide or transition metal complexes) from emission of short-lived fluorophores (e.g., organic dyes or proteins). This is particularly advantageous for applications such as security and biosensing^37^. Time-gating of SM-CPL spectrometer acquisition has previously been achieved and used by others to study chiral molecular interactions^45–49^, but the required SM-CPL spectrometer modifications were technically challenging and resulted in severe restrictions with regards to waveband scan range, poor acquisition rate, and poor signal to noise ratios^50,51^. Here, we achieve full-spectra time-gated detection with the SS-CPL spectrometer. For standard SS-CPL spectrometer acquisition, LED excitation is continuous and SS spectrometer detector integration time is variable. For the time-gated operation, a master trigger signal (43 Hz) is used to synchronise pulsed LED excitation and SS spectrometer detector integration time is fixed to 10 milliseconds. Time-gating is introduced by simply introducing a delay into the trigger signal for the SS spectrometer detectors (see Supplementary Fig. 2).

**Fig. 3:**
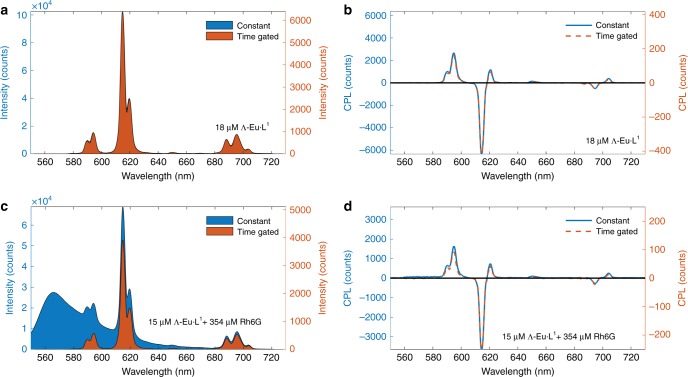
Time-gated detection enables accurate recovery of total intensity and CPL spectra of long-lifetime lanthanide emission from ~18 µM Λ-Eu·L^1^ in a 50:50 solution of MeCN and MeOH Eu·L^1^ (*τ* ~ 800 µs) in the presence of short-lifetime non-polarised fluorescence emission of ~350 µM Rh6G (*τ* ~ 4 ns). **a** The total emission measured by both constant and time gated modes is equivalent when there is no background fluorescence. **b** CPL of Λ-Eu·L^1^ is recovered by both constant and time-gated modes is equivalent when there is no background fluorescence. **c** Time-gating removes non-polarised fluorescent background from measurements of total intensity. **d** CPL is correctly recovered by both constant and time-gated modes in the presence of non-circularly polarised background fluorescence. Samples excited at 365 nm and spectra were recorded with standard scan settings. Note that the concentration of Λ-Eu·L^1^ is slightly decreased from **a**, **b** to **c**, **d** due to dilution.

Fig. 3 demonstrates recovery of total intensity and CPL spectra from Eu·L^1^ (emission lifetime, *τ* = 806 ± 5 µs) in solution with Rhodamine 6G (Rh6G), a non-polarised fluorophore (*τ* = 4.16 ns in H_2_O)^52^. The results in Fig. 3 demonstrate that whilst time-gated detection is not strictly necessary to recover accurate CPL spectra from Eu·L^1^, time-gated detection is essential to correctly recover total intensity (i.e., *I*_L-CPL_ + *I*_R-CPL_) of Eu·L^1^. Therefore, without time-gated detection, accurate *g*_em_ values for Eu·L^1^ would not be recovered in the presence of short-lived non-polarised background fluorescence.

## Discussion

We have demonstrated a novel SS-CPL spectrometer, and validated it’s performance and capabilities by direct comparison to a conventional SM-CPL spectrometer, which itself has been utilised in many prior peer-reviewed studies^10,21,22,24,36–41^. The SS-CPL spectrometer is capable of unprecedented real-time full-spectra CPL spectra acquisition, in as little as 10 milliseconds by using SS spectrometer detectors. Real-time SS-CPL operation was achieved by sample emission into two spatially separated samples: one detection channel for L-CPL and one for R-CPL (see the Methods section for full details). This SS-CPL (estimated component cost ~£12,000 at time of writing) affords advantages over conventional SM-CPL spectrometers; components for an in-house built SM-CPL system are estimated to cost ~£50,000 at the time of writing, whereas a commercially available SM-CPL costs over £100,000. Moreover, the modular design of the SS-CPL spectrometer can be potentially miniaturised and its rapid full-spectra time-gated detection capability renders it considerably more versatile.

Two chiral europium complexes were utilised as references in this study to compare the SS-CPL and SM-CPL spectrometers: (1) Eu·L^1^, a complex with modest *g*_em_ (ranging from 0.05 to 0.25), with favourable bright emission, two enantiomers, and a feature-rich CPL spectra, ideal for inter-instrument comparison^42^. (2) Eu{(+)-facam}_3_ a complex with unfavourable emission properties, a single available enantiomer, but a high *g*_em_ ~ −0.8, ^5^D_0_→^7^F_1_). However, there is no consensus on the exact *g*_em_ values for Eu{(+)-facam}_3_ between studies (refer to the Results section for further details). An alternative in-house synthesised inter-study reference complex could have been cesium tetrakis(3-heptafluoro-butylryl-(+)- camphorato) europium (III) (Cs[Eu((+)-hfbc)_4_]), which has two enantiomers, and the highest reported *g*_em_ to date: ^5^D_0_→^7^F_1_ (595 nm) *g*_em_ = 1.38, (2 mM in ChCl_3_)^19,53^. Several studies have confirmed *g*_em_ of Cs[Eu((+)-hfbc)_4_, making it a desirable reference complex for future benchmarking of CPL spectrometers^25,54–57^. Rapid acquisition of CPL spectra with the SS-CPL spectrometer will be most advantageous for increasing SNR in CPL measurements, where noise in CPL measurement and related uncertainty in estimates of *g*_em_ fundamentally arises from photon shot noise, which is proportional to the square root of the total emission intensity. A strategy to improve SNR is to average many spectra, which reduces noise proportionally to the number of spectra averaged. Using standard scan-settings, the SM-CPL spectrometer used in this study requires around 45 min to accumulate 5 full spectra (570–720 nm). In comparison, the SS-CPL spectrometer can acquire 5 full spectra (400–800 nm) in as little as 50 milliseconds: approximately 5400 times faster than the SM-CPL spectrometer. Further, accumulation of many repeated spectra enable uncertainty in *g*_em_ to be accurately and conveniently calculated by quantifying fluctuations in intensity of L-CPL and R-CPL channel intensity measurements (see Supplementary Note 3) Such direct uncertainty quantification will be an improvement over many SM-CPL spectrometer studies, which typically report an assumed nominal uncertainty rather than an uncertainty from repeated measurement. For example, when studying the ^5^D_0_→^7^F_4_ transition of Eu:BPEPC, Bonsall et al.^43^ reported *g*_em_ = 10^−3^ ± 10^−2^, i.e., a nominal uncertainty an order of magnitude greater than the estimated value of *g*_em_.

Whilst we have demonstrated successful measurement of high *g*_em_ CPL emission with a SS-CPL spectrometer, it is essential to consider how minor systematic instrumental errors may influence recovery of CPL spectra, particularly in the regime of *g*_em_ ⩽ 10^−2^, where CPL is fundamentally challenging to detect. Firstly, the performance of the achromatic QWP used in the SS-CPL must be considered. With a retardance of 0.25 waves, a perfect achromatic QWP theoretically converts all circularly polarised light into linearly polarised light using linear polarisers prior to detection. However, deviation of retardance from 0.25 waves (due to manufacturer specification or trivial off-axis misalignment) will result in circularly polarised light being converted into light with some degree of elliptical polarisation, which will be partly transmitted through the linear polariser analysers, resulting in a small over-estimate of CPL signal. This is not problematic for SM-CPL spectrometers because a PEM can be calibrated to provide wavelength-specific uniform retardance. However, achromatic QWPs exhibit minor variations in retardance across wide wavelength range: e.g., the achromatic QWP employed in the SS-CPL spectrometer has a retardance of 0.25 waves specifically at ~450 nm and ~750 nm (manufacturers specifications). Any errors arising due to non-uniform QWP retardance were not noticeable in this study of high *g*_em_ CPL spectra over a fairly constrained wavelength range (570–720 nm). However, further consideration to mitigate this potential systematic error is prudent if the SS-CPL spectrometer is to be applied to measurement of wide-band, low *g*_em_ CPL measurements typical of organic molecules such as BODIPYs (see Table 1). Mitigation strategies could include use of cage-mount to minimise trivial QWP alignment errors; constraining measurement wavebands to areas of optimal achromatic QWP retardance; or utilizing a super-achromatic QWP. A second source of systematic error arises from the orientation and alignment of the linear polariser analysers. Off-axis misalignment will degrade extinction ratio of the linear polarisers, thereby resulting in an over-estimate of CPL signal; such trivial misalignment could be mitigated by using cage mount. More fundamentally, the motorised rotation mounts used to control the orientation of the linear polariser analysers in the SS-CPL spectrometer have a bidirectional repeatability of ±0.1°, which will result in minor differences in repeated measurement of R-CPL and L-CPL channels. These minor variations will be exacerbated for real-time acquisition, where the dual channel intensity-matching procedure is employed. Such pseudo-random variabilities could be quantified by repeated measurements of a reference sample. Further, uncertainties arising from dual-channel operation could be eliminated by acquiring CPL measurements with only a single SS-CPL spectrometer detection arm; whilst this would reduce CPL spectra acquisition rate, a SS-CPL spectrometer operating with a single-arm operation would still be able to acquire CPL spectra very rapidly, e.g., in around 10 seconds, because it would only be limited by the rotation speed of the motorised rotation mounts. This is obviously a trade-off by demoting the instrument form simultaneous L- and R-CPL detection. Furthermore, the lack of an accepted reference standard for low *g*_em_ prevents us from further validating our instruments limits and performance in this regime. Overall, these potential sources of systematic errors mandate further exploration, via experiment and/or optical simulation, to assess the minimum *g*_em_ values that can be reliably measured with a SS-CPL spectrometer.

The SS-CPL spectrometer design is fundamentally modular in nature and can be upgraded or modified in future to provide enhanced capabilities. We envisage several alternative SS-CPL configurations, depending on end-user application. For example, the single UV excitation LED light source used here could be substituted for more versatile light sources, such as an array of multiple monochromatic LEDs^58^, a pulsed arc lamp, or a tuneable modulated laser light source to accommodate a wider range of excitation wavebands. Fibre-optics could be used to miniaturise the design and deliver light to alternative detectors, e.g., to a single CCD detector array, reducing the requirement to compensate for non-identical performance of two individual SS CCD detectors. Further miniaturisation could be achieved by using miniature SS CCD spectrometers (e.g., <3 cm in size)^35^, enabling the prospect of portable CPL spectroscopy. Beyond spectroscopy, the core optical design of the SS-CPL spectrometer could be adapted for use with a laser scanning confocal microscope (LSCM) to enable chiral confocal microscopy^37^. With prospects of rapid acquisition, time-gated full-spectra detection, and miniaturised low-cost instrumentation, it is possible to envisage the use of SS-CPL spectrometers for high-throughput wavelength-multiplexed chiral screening, e.g. for lanthanide-based biosensing or security applications^37,59,60^.

In conclusion, we have demonstrated a SS-CPL spectrometer design using a novel dual-channel optical layout and fast-readout SS-CCD spectrometer detectors to enable time-resolved whole-spectra CPL measurements of chiral europium complexes (*g*_em_ > 0.05) in as little as 10 milliseconds. This is an unprecedented improvement in capabilities over conventional SM-CPL spectrometers, which are expensive to build and maintain, typically require tens of minutes to acquire a full CPL spectrum, and which have remained fundamentally unchanged for over 50 years. In contrast, the SS-CPL spectrometer reported here does not share these limitations. In theory, SS-CPL spectrometers should be broadly applicable to measurement of a wide variety of CPL emitters, not just lanthanide complexes. However, further collaborative investigation of potential minor systematic errors arising from hardware limitation is required before the SS-CPL spectrometer can be applied to CPL measurement of low *g*_em_ emitters (*g*_em_ ≤ 10^−2^), such ketones, helicenes, BODIPYs, etc. We anticipate that the new measurement capabilities and convenient design realised by SS-CPL spectrometers will remove barriers to entry and promote wider uptake and dissemination of CPL spectroscopy.

## Methods

### SM-CPL spectrometer design and operation

A custom-built SM-CPL spectrometer was used as a direct comparison for the SS-CPL system. This SM-CPL spectrometer has been utilised in many prior studies^10,21,22,24,36–41^ and full technical details of its design are reported by Carr et al.^33^. The emission monochromator slit width was adjusted so that the full width half maximum (FWHM) of atomic emission lines from a neon light source measured by the SM-CPL spectrometer closely matched the FWHM of the same lines measured by the SS-CPL spectrometer, ensuring that emission peak line width, line shape, and peak position were as closely matched as possible between the two CPL spectrometers. Standard scan range for europium samples are 570–720 nm in 0.5 nm scan increments with 500 µs integration time per increment, requiring a total of 45 min to acquire 5 accumulated scans; however, for this study, 0.2 nm scan increments were used in order to closely match the sampling increments of the SS-CPL spectrometer, requiring a total of 112 min to acquire 5 accumulated scans.

### SS-CPL spectrometer design and operation

The SS-CPL spectrometer set up is shown in Fig. 1b (see also Supplementary Fig. 1). Sample excitation was provided by a 2.35 W 365 nm LED with a 9 nm spectral half-width (CUN6AF4A; Roithner LaserTechnik) mounted in a custom-built heat sink. Power to the LED was supplied by a bench-top power supply (PL303QMD 30V/3A; Aim TTI) operating in constant current mode. This builds upon the work of Castiglioni et al.^58^ who demonstrated the utility of LED light sources in CPL spectrometers. A collimating lens, ground-glass diffuser (N-BK7, Thorlabs), and a 240–395 nm bandpass filter (FGUV5, Thorlabs) placed prior to the sample ensured that excitation light was diffuse, unpolarised, and constrained to *λ* < 400 nm. Samples were placed within an enclosed holder (CVH100, Thorlabs). All samples were measured in quartz cuvettes with a 10 mm by 10 mm cross-section (111-10-40; Hellma Analytics). Sample emission was collected 90° to the excitation beam. The emitted light passed through an achromatic QWP (AQWP05M-600; Thorlabs), which converted circularly polarised light into two orthogonal, linearly polarised, signals, corresponding to L-CPL and R-CPL. The light then was then split into two spatially separated detection channels by a non-polarising 50/50 beam splitter (BS013; Thorlabs). Each detection channel was capable of independently measuring L-CPL and R-CPL in a sequential manner by precisely rotating a linear polariser (LPVISE100-A; Thorlabs) mounted in a motorised precision rotation mount (PRM1/MZ8; Thorlabs) controlled via an electronic controller [KDC101; Thorlabs] for automated and repeatable positioning of the linear polariser (see Supplementary Note 1). A long pass filter (*λ* > 450 nm) (FEL0450, Thorlabs] prior to the CCD detectors ensured no stray excitation light could reach the detector. Emission intensities of L-CPL and R-CPL channels were quantified by two matched high-sensitivity SS CCD spectrometer operating at 400–800 nm with ~0.2 nm sampling increments (Maya2000Pro, H3 grating, 350–850 nm, Ocean Optics). To maximise light throughput to the CCD detector, the spectrometer entrance aperture slit was removed (see Results and Discussion sections for rationale).

From the intensity of each channel, the CPL spectra were calculated by: ${\mathrm{CPL}}\left( {\uplambda} \right) = I\left( {\uplambda} \right)_{{\mathrm{L}} {\hbox{-}} {\mathrm{CPL}}}\, -\, I\left( {\uplambda} \right)_{{\mathrm{R}} {\hbox{-}} {\mathrm{CPL}}},$ and $g_{{\mathrm{em}}}\left( {\uplambda} \right) = \frac{{2\left[ {I\left( {\uplambda} \right)_{{\mathrm{L}} {\hbox{-}} {\mathrm{CPL}}} - I\left( {\uplambda} \right)_{{\mathrm{R}} {\hbox{-}} {\mathrm{CPL}}}} \right]}}{{\left[ {I\left( {\uplambda} \right)_{{\mathrm{L}} {\hbox{-}} {\mathrm{CPL}}} + {\mathrm{I}}\left( {\uplambda} \right)_{{\mathrm{R}} {\hbox{-}} {\mathrm{CPL}}}} \right]}}$ (refer also to Supplementary Eq. 4). Synchronised operation of both channels enabled rapid concurrent acquisition of full CPL spectra. This also required a dual-channel intensity-matching procedure derived in situ for each sample measured (see Supplementary Note 2). SS-CPL operation was automated using custom-written LabVIEW programs (LabVIEW 2013, National Instruments). Data was analysed post-hoc with custom-written Matlab scripts (Matlab 2019a, MathWorks).

Time-resolved detection of whole-spectra was achieved by introducing a time delay between the pulsed LED excitation and the start of SS CCD spectrometer acquisition (see Supplementary Fig. 2) using synchronised transistor–transistor logic (TTL) signals produced via a USB multifunction I/O device (USB-6210, National Instruments). Acquisition at a rate of 43 Hz was found to minimize spurious noise (e.g., from mains frequency electronics at 50 Hz) whilst providing a suitable measurement window for time-resolved measurement of long-lived lanthanide emission (i.e., several milliseconds) with a 10-millisecond integration time.

### SS-CPL spectrometer calibration

For the SS-CPL to correctly and rapidly measure CPL spectra, it was necessary to perform two calibration procedures: (1) calibration of the linear polariser rotation angle to acquire optimal CPL spectra (only necessary when first aligning the SS-CPL spectrometer). (2) A dual-channel intensity-matching calibration to match the emission intensity recorded by each detection channel, accounting for variables such as differences in the alignment of optical components, as well as the wavelength-dependent sensitivity of each SS CCD spectrometer, thereby enabling equivalent concurrent dual-channel measurements of L-CPL and R-CPL. This step was performed with the sample of interest in situ.

Linear polariser rotation angle calibration: this procedure was necessary when first aligning the SS-CPL spectrometer. An automated scanning routine was used to ascertain the linear polariser rotation angles required to measure L-CPL and R-CPL. This was achieved by first measuring a feature-rich CPL spectra (e.g., that of an enantiomer of Eu·L^1^) with the SM-CPL spectrometer to serve as a ground truth reference. The sample was then loaded into the SS-CPL spectrometer and the SS-CPL linear polarisers were rotated 360° in 1° stepwise increments, with a spectrum recorded at each increment. From this data 360 separate candidate CPL spectra were produced and compared against the reference SM-CPL spectra by use of a comparison metric (described in the Supplementary Note 1). Near-optimal CPL measurement was achieved when this comparison metric was minimised. This process was repeated at 0.1° stepwise increments at ±1° from the approximate optimal linear polariser rotation angles. More accurate adjustments to linear polariser rotation angle were achieved by measuring the response of the SS-CPL spectrometer to a non-polarised fluorescent emitter (e.g. Rhodamine 6G); correct orientation of linear polariser rotation angle was achieved when apparent CPL signal recovered was nulled. Once the correct linear polariser rotation angles were determined, the linear polariser could be simply rotated ±90° to analyse L-CPL and R-CPL states selectively.

Dual-channel intensity matching calibration. Prior to the measurement of each sample, the sample of interest was placed inside the cuvette holder. Each detection channel was set to measure L-CPL. From this data, a multiplicative correction factor to match the emission intensity recorded in each channel was derived. The multiplicative correction factor for each sample was then applied to subsequent measurements of that sample, enabling rapid acquisition of CPL spectra (see Supplementary Eq. 4).

## Supplementary information

Supplementary Information

## Data Availability

The data generated and analysed during this study are available from the corresponding author upon reasonable request.
