# Supplementary material for: Rapid time-resolved Circular Polarization Luminescence (CPL) emission spectroscopy
Source: Nat Commun. 2020 Apr 3;11:1676. doi: 10.1038/s41467-020-15469-5 (PMC7125171; doi:10.1038/s41467-020-15469-5)
Supplement: Supplementary file 1 — Supplementary Information [file 41467_2020_15469_MOESM1_ESM.pdf]

Rapid Time-Resolved Circular Polarization Luminescence (CPL) Emission Spectroscopy

Supplementary Information

Lewis E. MacKenzie, Lars-Olof Pålsson, David Parker, Andrew Beeby, and Robert Pal.*

Department of Chemistry, Durham University, South Road, Durham, United Kingdom. DH1 3LE.

*Corresponding author: robert.pal@durham.ac.uk

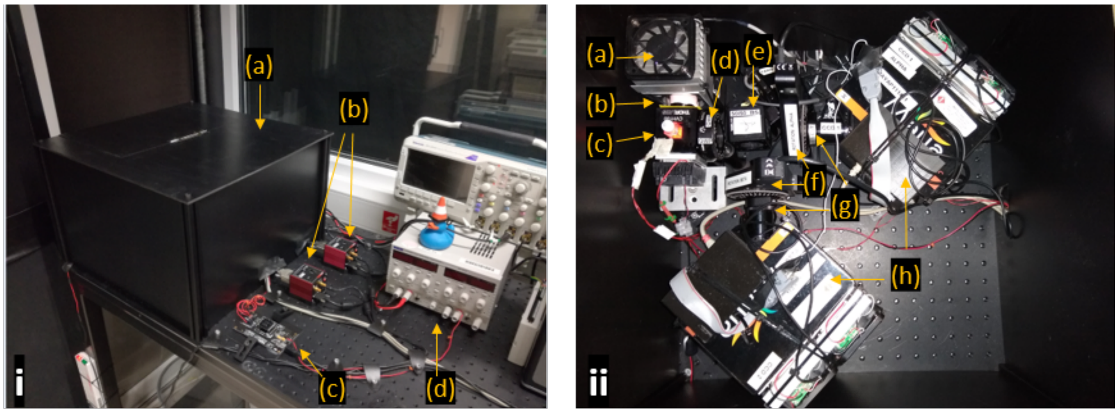

Supplementary Figure 1. Left: bench top view of the SS-CPL spectrometer. (a) SS-CPL spectrometer within light-proof enclosure on laboratory bench (enclosure dimensions 48 x 48 x 30 cm); (b) linear polarizer rotation mount control units; (c) cuvette temperature control unit electronics (not utilized in this study); (d) bench-top LED power supply. **Right: The SS CPL spectrometer optical components.** (a) UV Excitation LED (365 nm, 9 nm FWHM) in a custom heat-sink mount; (b) collimating lens, diffuser, and λ < 395 nm short pass filter; (c) Cuvette holder with temperature control components. A europium complex is present in the glass cuvette. (d) QWP in rotation mount; (e) 50/50 non-polarizing beam splitter cube in positioning mount; (f) Linear polarisers in automated motorized rotation mounts; (g) λ > 450 nm long-pass filters. (h) SS CCD spectrometer detectors mounted on height-adjustable lab jacks. Component model listings are provided in the methods section.

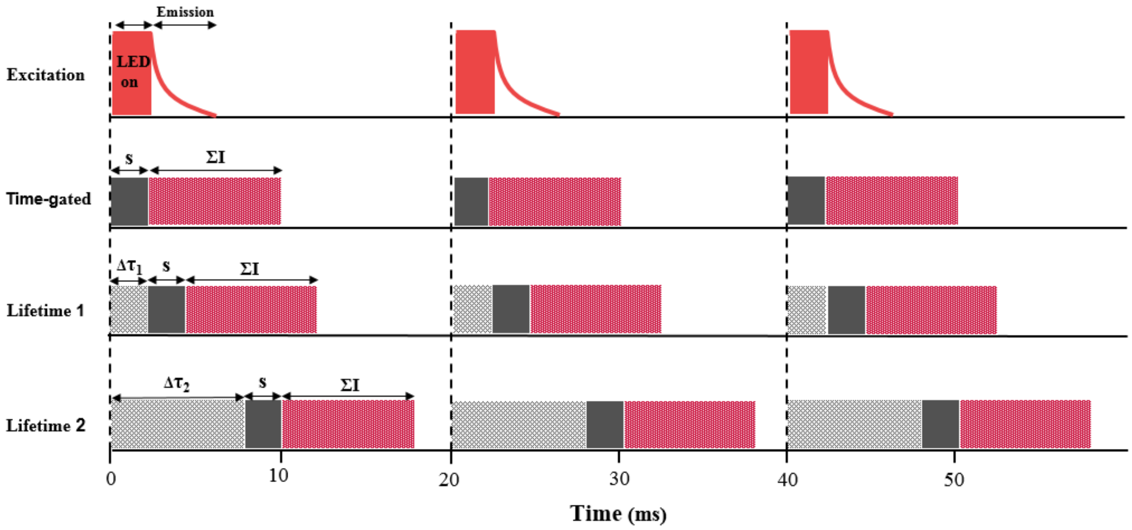

Supplementary Figure 2. The principle of time-gated detection and extension to time-resolved measurement of emission lifetimes in the 10 µs – 10 ms regime. ΣI = spectrometer integration time; s = CCD activation time; Δτ = variable delay time. In this example, a cycle frequency of 50 Hz is a shown but in practice a 43 Hz cycle frequency was utilized to avoid introducing noise arising from UK mains-frequency electricity at 50 Hz.

Supplementary Note 1.

SS-CPL calibration: alignment of linear polariser rotation angle to recover L-CPL and R-CPL and adjustment of beam splitter position

An automated scanning algorithm was used to ascertain which linear polariser rotation angle corresponded to L-CPL and R-CPL for each detection channel with sub-degree precision. Firstly, a 'ground truth' reference CPL spectra was obtained by measuring CPL emission of a feature-rich sample (e.g. Λ-Eu·L[1]) with the SM-CPL spectrometer. This reference sample was then loaded into the SS-CPL spectrometer. Each detection channel linear polariser was then automatically rotated through 360 degrees in 1° increments and a spectrum was recorded at each increment. At each angle, hereby referred to as 'the primary angle', a candidate CPL spectrum was calculated by subtracting the spectrum recovered at the primary angle from the spectrum recovered at an angle +90° to the primary angle (hereby referred to as the 'secondary angle'). A comparison metric was then calculated to compare the similarity between the 'ground truth' refence CPL spectra and candidate CPL spectra. This comparison metric was the sum of the residuals obtained by subtracting the reference CPL spectra from each candidate CPL spectrum. Correct approximate rotation of the linear polarisers (i.e. an alignment of ± 1°) was achieved when the comparison metric was minimised (see Supplementary Figure 3). Subsequently, the position of the 50/50 beam splitter was manually adjusted by using a kinematic mount to further minimise the comparison metric for both channels.

The linear polariser rotation angle was then further refined by rotating the polariser ± 1° around the prior primary angle in 0.1° increments and recalculating the comparison metric as appropriate. The final calibration step was calibration to a bright non-polarised fluorescence emitter. In this calibration mode, when the linear polarisers are correctly aligned, the difference between L-CPL and R-CPL should be zero by definition. However, if the linear polarisers are not optimally orientated, then non-CPL fluorescence will result in a deviation from null signal. In this respect, non-polarised

calibration of linear polariser rotation angle is more sensitive than calibration to a 'ground truth' CPL reference spectra, and therefore can provide a more precise calibration. In theory, any arbitrary fluorophore with non-circularly polarised fluorescence emission could be used. However, for this study, 40 μM Rhodamine 6G in H_2O was used as a non-circularly polarised emission standard. The sample was contained within a plastic cuvette, which served to eliminate any residual polarization effects via multiple scattering of emitted photons. The linear polariser primary angle was then adjusted in 0.1° increments until the recovered CPL signal was nulled. The precise linear polariser primary angle was then subsequently utilized as the linear polariser rotation angle for L-CPL, with the angle corresponding to R-CPL by definition orthogonal (90°) to the L-CPL channel.

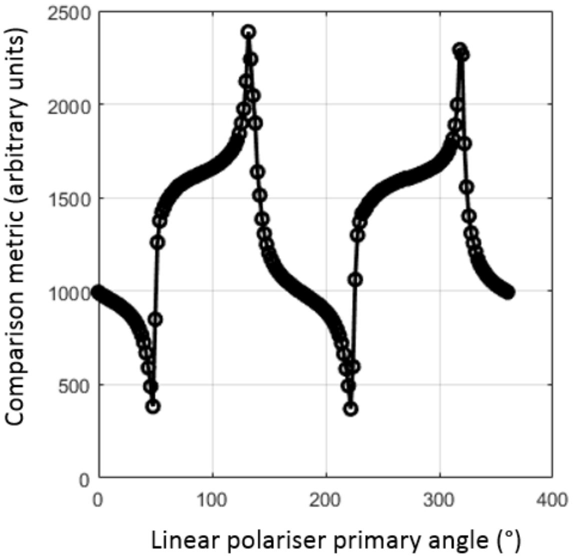

Supplementary Figure 3. Calibrating linear polariser rotation angle to recover CPL. A comparison metric was used to compare a 'ground truth' CPL spectra to each candidate CPL spectrum; near-optimal CPL measurement is achieved where the comparison metric is minimised, e.g. at linear polariser rotation angles of ~50° and ~130° in the above example. This approximate alignment was then further refined by (a) scanning in smaller angular steps (0.1°), and (b) by aligning to ensure that null CPL signal is recovered when measuring non-polarised emission.

Supplementary Note 2:

SS-CPL calibration: detection channel intensity-matching procedure

To achieve simultaneous detection of L-CPL and R-CPL with the two spatially separated detection channels, it is necessary to account for the fact that both detection channels record equivalent, but non-identical measurements of intensity due to differences in component alignment and inter-detector responses. Therefore, an intensity-matching factor is required.

First, the sample to be measured is loaded into the SS-CPL spectrometer. Then both detection channels are an equivalent signal, i.e. total intensity of L-CPL. From this measurement, a wavelength-dependent *in-situ* multiplicative intensity correction factor, $\mathrm{CF}(\lambda)$, is derived:

$$\mathrm{CF}(\lambda) = \frac{\mathrm{IA}(\lambda)_{\text{L-CPL}}}{\mathrm{IB}(\lambda)_{\text{L-CPL}}}. \qquad \text{(Supplementary Equation 1)}$$

Where $\mathrm{IA}(\lambda)_{\text{L-CPL}}$ and $\mathrm{IB}(\lambda)_{\text{L-CPL}}$ is the intensity measured by each spatially separated measurement channel (denoted as A and B respectively) when set to measure L-CPL. For subsequent measurements, the intensity recorded by channel B is adjusted by $\mathrm{CF}(\lambda)$:

$$\mathrm{IB}(\lambda)_{\mathrm{ArbCPL}}^{\mathrm{Corrected}} = \mathrm{IB}(\lambda)_{\mathrm{ArbCPL}}{*}\mathrm{CF}(\lambda). \qquad \text{(Supplementary Equation 2)}$$

Where $\mathrm{IB}(\lambda)_{\mathrm{ArbCPL}}$ is the intensity measured by channel B when the channel is set to measure an arbitrary CPL state, and $\mathrm{IB}(\lambda)_{\mathrm{ArbCPL}}^{\mathrm{Corrected}}$ is the resulting corrected intensity. The CPL spectra, $\mathrm{CPL}(\lambda)$, is then:

$$\mathrm{CPL}(\lambda) = \mathrm{IA}(\lambda)_{\text{L-CPL}} \text{-} \mathrm{IB}(\lambda)_{\text{R-CPL}}^{\mathrm{Corrected}}. \qquad \text{(Supplementary Equation 3)}$$

And that $g_{\mathrm{em}}(\lambda)$ is then calculated by:

$$g_{\mathrm{em}}(\lambda) = \frac{2\left(\mathrm{IA}(\lambda)_{\text{L-CPL}} \text{-} \mathrm{IB}(\lambda)_{\text{R-CPL}}^{\mathrm{Corrected}}\right)}{\left(\mathrm{IA}(\lambda)_{\text{L-CPL}} + \mathrm{IB}(\lambda)_{\text{R-CPL}}^{\mathrm{Corrected}}\right)}. \qquad \text{(Supplementary Equation 4)}$$

Supplementary Note 3:

SS-CPL: estimation of uncertainty in g_{em}

To derive the uncertainty associated with our estimation of emission dissymmetry factor, Δg_{em}, it is first necessary to ascertain the uncertainty associated with $I_{\text{L-CPL}}$ and $I_{\text{R-CPL}}$. At the most fundamental level, uncertainty in intensity measurements arises from shot noise, i.e. random fluctuations in the emission rate of photons from the sample, proportional to the square root of the number of emitted photons.[1] We use the standard error, (σ), of mean intensity measured by each channel as a measure of the uncertainty in $I_{\text{L-CPL}}$ and $I_{\text{R-CPL}}$.

To begin, we start with the definition of g_{em}:

$$g_{em} = \frac{2a}{b}, \qquad \text{(Supplementary Equation 3)}$$

$$a = \left(I_{\text{L-CPL}} - I_{\text{R-CPL}}\right),$$

$$b = I_{\text{L-CPL}} + I_{\text{R-CPL}}.$$

Where $I_{\text{R-CPL}}$ and $I_{\text{R-CPL}}$ are the mean total intensity recorded for L-CPL and R-CPL. The relative uncertainties in a and b $(\Delta a, \Delta b)$ can be estimated by considering the standard error of the mean total intensity recorded for L-CPL and R-CPL, denoted as $\sigma_{\text{L-CPL}}$ and $\sigma_{\text{R-CPL}}$:

$$\Delta a = \sqrt{\left(\frac{\sigma_{\text{L-CPL}}}{I_{\text{L-CPL}}}\right)^2 + \left(\frac{\sigma_{\text{R-CPL}}}{I_{\text{R-CPL}}}\right)^2}, \qquad \text{(Supplementary Equation 4)}$$

$$\Delta b = \sqrt{\left(\frac{\sigma_{\text{L-CPL}}}{I_{\text{L-CPL}}}\right)^2 + \left(\frac{\sigma_{\text{R-CPL}}}{I_{\text{R-CPL}}}\right)^2}.$$

Therefore, from Supplementary Equation 5, the relative uncertainty in emission dissymmetry factor (Δg_{em}), can be estimated by:

$$\Delta g_{\mathrm{em}} = 2\sqrt{(\Delta a)^2 + (\Delta b)^2}$$
$$= 2\left(\sqrt{\left(\left(\frac{\sigma_{\text{L-CPL}}}{I_{\text{L-CPL}}}\right)^2 + \left(\frac{\sigma_{\text{R-CPL}}}{I_{\text{R-CPL}}}\right)^2\right) + \left(\left(\frac{\sigma_{\text{L-CPL}}}{I_{\text{L-CPL}}}\right)^2 + \left(\frac{\sigma_{\text{R-CPL}}}{I_{\text{R-CPL}}}\right)^2\right)}\right) \qquad \text{(Supplementary Equation 5)}$$

$$\rightarrow \Delta g_{\mathrm{em}} = 4\sqrt{\left(\frac{\sigma_{\text{L-CPL}}}{I_{\text{L-CPL}}}\right)^2 + \left(\frac{\sigma_{\text{R-CPL}}}{I_{\text{R-CPL}}}\right)^2}.$$

Given that standard error ($\sigma_{\text{L-CPL}}$ and $\sigma_{\text{R-CPL}}$) is calculated as the standard deviation of the mean total intensity for a given channel divided by the square root of the number of spectra acquired, Δg_{em} will be reduced by increasing the number of spectra averaged and/or maximising emission to minimise the relative influence of shot noise.

Supplementary Note 4:

SS-CPL spectrometer scan settings:

SS-CPL Constant excitation mode: SS CCD integration time was adjusted between 10 and 200 ms as appropriate to ensure a strong signal in the SS CCD detectors without detector saturation (e.g. ~45,000 counts out of a maximum 65,535 per SS CCD detector). Five spectra were accumulated and averaged *in-situ* and saved. Twenty spectra were saved and used for analysis.

SS-CPL Time-gated excitation mode: integration time was fixed at 10 ms per acquisition. Acquisition rate was 43 Hz. 50 spectra were accumulated and averaged *in-situ* and 20 spectra were saved and used for analysis.

Supplementary Note 5:

Data processing:

SS-CPL data processing steps: 20 saved spectra were averaged. Then the constant baseline present in the measurements was estimated by a linear fit to wavelengths of zero emission, and then subtracted from readings. The *in-situ* multiplicative intensity correction factor, $\mathrm{CF}(\lambda)$, was then applied to the intensity measurements of channel B (see Supplementary Equation 2). An instrument response correction derived from measurements of a standard reference lamp (HL2000-CAL, Ocean

Optics) was then applied to measurements of both channel A and channel B. The resultant CPL spectra, g_{em}, and Δg_{em} were then calculated as per Supplementary Equations 3, 4, and 7.

SM-CPL data processing steps: total intensity was zeroed by subtraction of any baseline offset, which was estimated by a fit to intensity data from wavelengths corresponding to zero emission intensity. An instrument response correction derived from measurements of a standard reference lamp (HL2000-CAL, Ocean Optics) was then applied to total intensity (DC signal component) and CPL signal (AC signal component). g_{em} was calculated by $g_{\mathrm{em}} = \frac{2(\mathrm{CPL})}{\mathrm{Total\ intensity}}$.

Supplementary Note 6:

Emission lifetime measurements

The emission lifetime of Λ-Eu·L[1] was measured using a PerkinElmer LS55 spectrophotometer using the following scan settings: excitation: 365 nm; excitation slit width: 10 nm; emission: 610 nm; emission slit width: 10 nm; delay steps: 0.1 – 7 ms in 0.1 ms increments. Lifetime was estimated using a custom-written MatLab script which conducted a least-mean-squares fit to the data, modelled by an exponential decay and a constant offset. Reported uncertainties were calculated from 95% confidence bounds of this fit.

Supplementary Note 7:

Sample preparation

Eu·L^1

Λ-Eu·L^1 and Δ-Eu·L^1 were synthesized by Dr Matthieu Starck (Department of Chemistry, Durham University). Full details of Eu·L^1 synthesis is reported byn Starck et al., (2019).[2] 18 μM solutions of Eu·L^1 were prepared in a 50:50 mixture of MeCN and MeOH using absorption at 365 nm to estimate molar concentration. Samples were first measurement with the SS-CPL spectrometer then the SM-CPL spectrometer.

Eu{(+)-facam}$_3$

Europium tris[3-(trifluoromethylhydroxymethylene)-(+)-camphorate] [Eu{(+)-facam}$_3$] (CAS Number 34830-11-0) was supplied by Sigma-Aldrich. The compound was weighed using a high precision (0.1 mg) balance. Samples were prepared to 5.5 mM concentration in > 99.5% anhydrous DMSO (Dimethyl sulfoxide) [Sigma] immediately prior to measurement. The 5.5 mM concentration was selected so that results could be directly comparable with Brittain and Richardson (1976) and Sánchez-Carnerero et al., (2015).[1,3] However, emission intensity was fairly low compared to the sample of Eu·L^1 (~ 5 mV vs. ~300 mV, respectively). Samples were first measurement with the SS-CPL spectrometer then the SM-CPL spectrometer.

Rhodamine 6G

Rhodamine 6G (Rh6G) (CAS Number 989-38-8) was obtained from Sigma-Aldrich. The compound was weighed using a high precision (0.1 mg) balance. A 2 mM stock solution of Rh6G in a 50/50 mixture of MeCN and MeOH was prepared which was incrementally added to a solution of Λ-Eu·L^1 as

required to increase the intensity of emission from Rh6G to levels similar to that of Λ-Eu·L[1] (see Supplementary Figure 4).

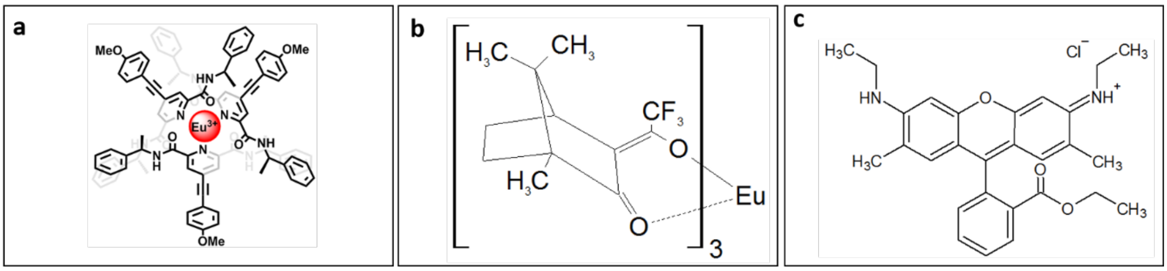

Supplementary Figure 4: chemical structures of relevant compounds. (a) Λ-Eu·L[1] (adapted from Supplementary Reference 2 by permission of The Royal Society of Chemistry).[2] (b) Eu{(+)-facam}$_3$. (c) Rhodamine 6G.

Supplementary References

1. Sánchez-Carnerero, E. M. *et al.* Circularly Polarized Luminescence from Simple Organic Molecules. *Chem. - A Eur. J.* **21**, 13488–13500 (2015).

2. Starck, M., Mackenzie, L., Batsanov, A. S., Parker, D. & Pal, R. Excitation modulation of Eu:BPEPC based complexes as low-energy reference standards for circularly polarised luminescence (CPL). *Chem. Commun.* (2019). doi:10.1039/C9CC07290J. Available online at: https://pubs.rsc.org/en/content/articlelanding/2019/cc/c9cc07290j#!divAbstract.

3. Brittain, H. G. & Richardson, F. S. Circularly Polarized Emission Studies on the Chiral Nuclear Magnetic Resonance Lanthanide Shift Reagent Tris(3-trifluoroacetyl-d-camphorato)europium(III). *J. Am. Chem. Soc.* **98**, 5858–5863 (1976).
